# A Thioredoxin Homologous Protein of Plasmodium falciparum Participates in Erythrocyte Invasion

**DOI:** 10.1128/IAI.00289-18

**Published:** 2018-07-23

**Authors:** Wei Wang, Peng Huang, Ning Jiang, Huijun Lu, Dongchao Zhang, Dawei Wang, Kai Zhang, Mats Wahlgren, Qijun Chen

**Affiliations:** a Key Laboratory of Zoonosis, Jilin University, Changchun, China; b Key Laboratory of Zoonosis, College of Veterinary Medicine, Shenyang Agricultural University, Shenyang, China; c Institute of Microbiology, Tumor and Cellular Biology Center, Karolinska Institutet, Stockholm, Sweden; University of South Florida

**Keywords:** erythrocyte, invasion, ligand, malaria, thioredoxin

## Abstract

Invasion of erythrocytes by merozoites is required in the life cycle of malarial parasites. Proteins derived from the invasive merozoites are essential ligands for erythrocyte recognition and penetration. In this study, we report a novel protein that possesses a Trx domain-like structure of the thioredoxin family and is expressed on the surface of merozoites of the malaria parasite Plasmodium falciparum. This protein, namely, PfTrx-mero protein, displayed a mutated sequence character at the Trx domain, but with a specific binding activity to human erythrocytes. Specific antibodies to the protein inhibited merozoite invasion into human erythrocytes. Immunization with a homologous protein of Plasmodium berghei strain ANKA also showed significant protection against lethal infection in mice. These results suggested that the novel PfTrx-like-mero protein expressed on the surface of merozoites is an important ligand participating in erythrocyte invasion and a potential vaccine candidate.

## Abstract (precis)

Invasion of erythrocytes by merozoites is required in the life cycle of malarial parasites. Proteins derived from the invasive merozoites are essential ligands for erythrocyte recognition and penetration.

## INTRODUCTION

Malaria, a severe infectious disease, is caused by parasites belonging to the genus *Plasmodium. Plasmodium falciparum*, the most virulent malarial parasite that infects humans, causes higher morbidity and mortality than other species (1). The emergence and wide dissemination of drug resistance strains of P. falciparum and insecticide-resistant mosquitoes propel the search for effective vaccines (2, 3). Merozoites are the invasive form of the parasite in the blood, and the infection is initiated by adherence to and penetration into erythrocytes (4). Blocking invasion by merozoites is an effective strategy for prevention of parasite infection (5). To date, a number of proteins expressed on the merozoite surface or associated proteins have been put into vaccination studies (6–10), though the clinical performance of the vaccine candidates has not been very satisfactory.

The process of erythrocyte invasion of a merozoite contains multiple receptor-ligand interactions. Several glycosaminoglycans (GAG), including sialic acid and heparin sulfate-like moieties on the surface of human erythrocytes, have been proved to be receptors for merozoite-derived proteins such as MSP-1 (11, 12). Recently, we revealed the heparin-binding proteome of P. falciparum (13). Apart from MSP-1, a number of proteins expressed at the early developmental stage of the parasite showed specific binding activity to heparin (13). These proteins all contain one or several GAG-binding motifs with a characteristic amino acid content such as “-X-B-B-X-B-X-” or “-X-B-B-B-X-X-B-X-,” where B is a basic residue such as lysine, arginine, or histidine (14, 15). Among the heparin-binding proteins, one of the proteins, encoded by PF3D7_1104400, attracted our attention. The amino acid sequence of the encoded protein showed high similarity to thioredoxin (Trx). The Trx family in Plasmodium contained three Trx proteins (Trx1, -2, and -3) and a number of Trx-like proteins. The Trx proteins are critical for maintaining the intracellular redox balance of the parasite and the infected erythrocyte (16), but the function of the Trx-like proteins is still speculative. The intracellular antioxidant function of thioredoxins is mediated by the conserved Cys-Gly-Pro-Cys motif (CGPC) (17). Additionally, there are three other noncatalytic cysteine residues in Trx, i.e., Cys62, Cys69, and Cys73 (18), among which Cys62 and Cys69 can form an additional disulfide bond to form a hydrophobic pocket, and the hydrophobic region often plays an important role in extracellular protein-protein interaction (19, 20). Further, most of the proteins in the Trx family function in the cytoplasm, but some of them have been found to be secreted by an unknown route and function extracellularly like protein binding (21, 22). For instance, the Trx-like protein in Trichuris suis has been reported to bind to intestinal epithelial cells, and a shorter form of Trx can be secreted outside the parasite and binds to the outer membrane of human U937 cells and MP-6 cells (23–25).

In this study, a novel protein encoded by PF3D7_1104400 was found to possess a conserved sequence feature of the Trx family. It was expressed mainly at the merozoite surface and participated in erythrocyte invasion by binding to heparin sulfate receptors on the erythrocytes. The data revealed a novel function of the Trx family proteins in the malaria parasite P. falciparum.

## RESULTS

### The amino acid sequence encoded by PF3D7_1104400 possesses a conserved thioredoxin domain.

**FIG 1:**
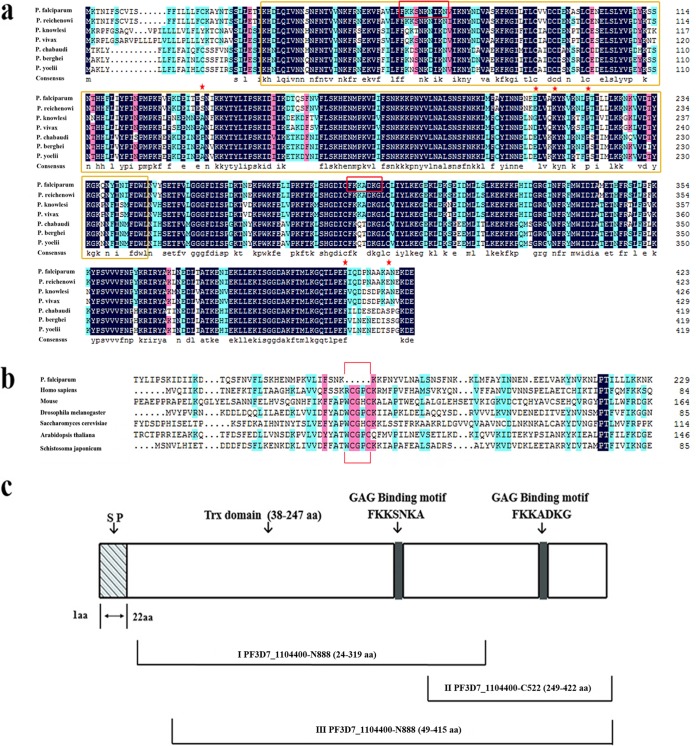
Sequence analysis and schematic representation of the protein encoded by PF3D7_1104400. (a) Sequence alignment of the PfTrx-like-mero protein with homologous proteins in other Plasmodium species. A predicted Trx domain is shown in the yellow box (amino acids [aa] 38 to 247), while two GAG-binding motifs are identified in a red box (residues FKKSNKA and FKKADKG). Red stars indicate the three conserved noncatalytic cysteine residues in the Trx domain. (b) Trx domain sequence alignment of the PfTrx-like-mero protein with other Trx proteins. A gap in the PfTrx-like-mero protein sequence due to the absence of the “-CXXC-” motif is labeled in the red box. (c) Schematic illustration of the PfTrx-like-mero protein (encoded by PF3D7_1104400). The PfTrx-like-mero protein consists of 424 amino acid residues with a predicted molecular weight of 49 kDa. A signal peptide (SP, aa 1 to 22) is shown in a box with diagonal lines. The two GAG-binding motifs are indicated with gray boxes. The three fragments (I, II, and III) for expression as recombinant proteins (PfTrx-like-mero protein-N888, PfTrx-like-mero protein-C522, PfTrx-like-mero protein-1101) are illustrated below the schematic.

The amino acid sequence of the protein encoded by PF3D7_1104400 identified in the heparin-binding proteome (13) was bioinformatically analyzed. Interestingly, the sequence was found to contain a conserved domain with a high similarity to the thioredoxin (Trx) family. However, the motif (“-CXXC-”), which determines the function of antioxidation, was missing, though the three noncatalytic conserved cysteine residues (Cys51, Cys55, and Cys62) were kept (Fig. 1a and b). Further, this sequence exhibited a considerable conservation in parasites from the genus Plasmodium, with up to 85.38% similarity. It is phylogenically more related to the annotated Trx-like proteins in other Plasmodium species than to the P. falciparum PfTlps (see Fig. S1 in the supplemental material). Additionally, the N terminus of the sequence contained a classical signal peptide domain (Fig. 1c), indicating that the protein is eventually secreted outside the parasite. Further, two GAG binding motifs were also identified in the molecule (Fig. 1a and c).

### The protein is expressed in the blood stage and localized on the surface of P. falciparum merozoites.

**FIG 2:**
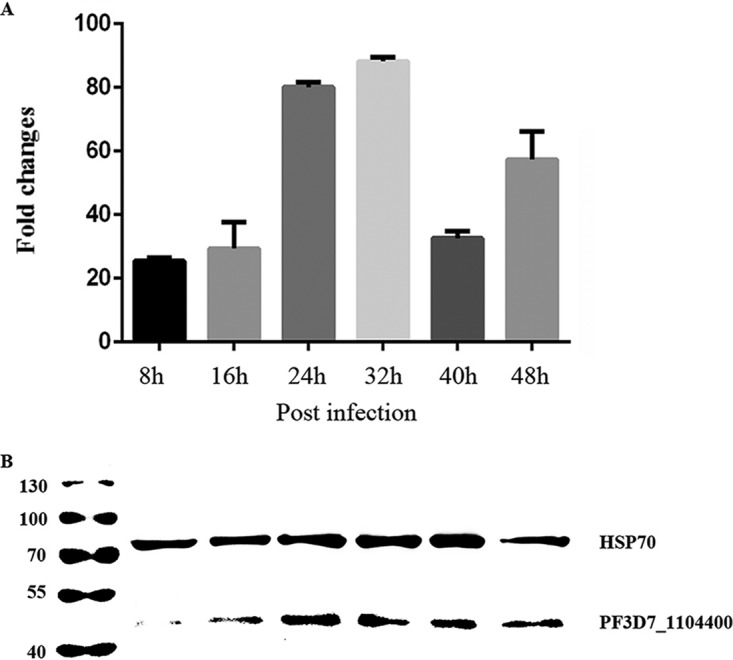
Transcription and expression of PF3D7_1104400 gene in the blood stage of P. falciparum strain 3D7. (A) Transcriptional analysis of the gene (PF3D7_1104400) by real-time PCR. The transcription levels of the gene at 8, 16, 24, 32, 40, and 48 h following erythrocyte invasion are shown. Results are the means and standard deviations from three separate experiments. (B) Expression the PfTrx-like-mero protein at the 6 time points was analyzed by Western blot assay. Heat shock protein 70 (HSP70) was used as an internal loading control.

The transcription and expression of the gene PF3D7_1104400 encoding the Trx-like protein during the blood stage of P. falciparum strain 3D7 were studied by real-time quantitative PCR (RT-qPCR) and Western blotting with synchronized parasites at 6 developmental time points following erythrocyte invasion. The results showed that the transcription and expression of the PF3D7_1104400 gene began early in the ring stage and reached a maximal level at 32 h (Fig. 2A and B).

**FIG 3:**
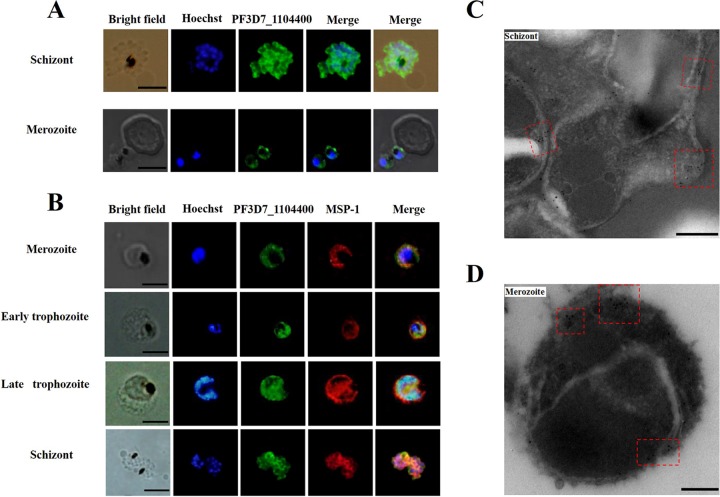
Distribution of the protein on the merozoite surface. (A) The distribution of the PfTrx-like-mero protein on merozoite in the schizont and before erythrocyte invasion was localized by indirect immunofluorescence. Nuclei are stained with Hoechst (in blue). The PfTrx-like-mero protein (encoded by PF3D7_1104400) is visualized by green fluorescence-labeled antibodies and was seen mostly on the periphery of the merozoites. (B) Colocalization of the PfTrx-like-mero protein with MSP-1 protein. The distribution of the PfTrx-like-mero protein is colocalized with MSP-1 during the asexual stage. After merozoite formation, the two proteins are distributed on the surface of both free and schizontic merozoites. At the trophozoite stage, the two proteins are distributed on the parasitophorous vacuole (PV) membrane and also inside the PV. Bar, 5 μm. (C and D) Localization of the PfTrx-like-mero protein by immune-electronic microscopy. Parasites at the late schizont stage (C) and free merozoites (D) were labeled using a protein-specific antibody and detected with a secondary antibody conjugated with gold particles. Gold particles are highlighted with red dashed frames. The protein is localized mostly on the merozoite surface. Bar, 200 nm.

Indirect immunofluorescence assays (IFA) revealed that the PfTrx-like-mero protein was expressed on the surface of both invading and schizontic merozoites colocalized with the MSP-1 protein (Fig. 3A and B). After merozoite invasion, this protein is distributed mostly inside the parasitophorous vacuole (PV) in a manner similar to that of MSP-1 (Fig. 3B). Further, in the immunoelectronic microscopy (IEM) assay with PfTrx-like-mero protein-specific antibodies, gold particles were clearly observed on the surfaces of both free and schizontic merozoites (Fig. 3C and D). Thus, the results of both IFA and IEM suggested that the PfTrx-like-mero protein is expressed on the surfaces of Plasmodium merozoites.

### The protein adhered to heparin and human erythrocytes.

**FIG 4:**
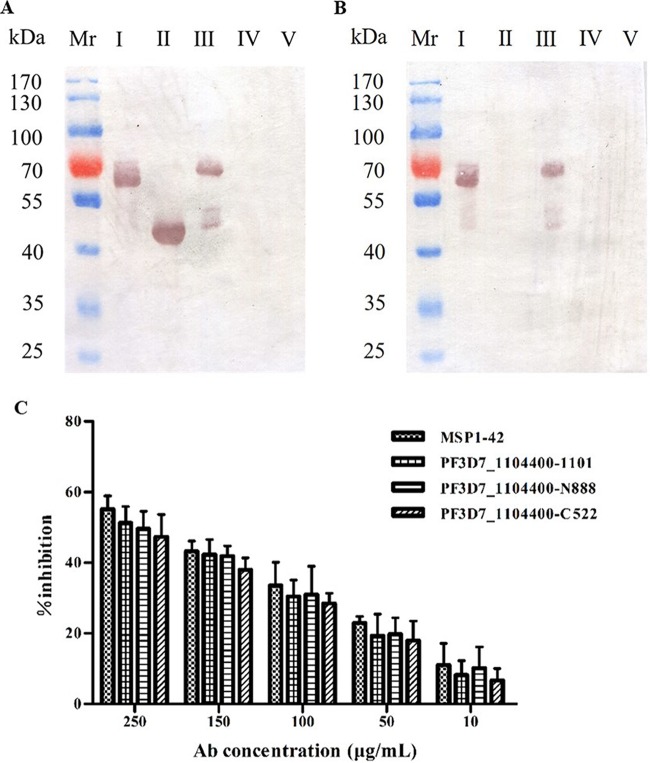
Association of the protein PF3D7_1104400 with merozoite invasion. (A) The recombinant PfTrx-like-mero proteins bind to heparin. The GST-tagged recombinant proteins of the three fragments of the PfTrx-like-mero protein (Fig. 1C and S2) were separately incubated with heparin-Sepharose, and their binding activity to heparin was detected by Western blotting. GST protein (lane IV) and uncoupled Sepharose (lane V) were used as controls. All three fragments bound to heparin but not to the Sepharose beads. GST did not show any binding to heparin. (B) Adhesion of recombinant PfTrx-like-mero proteins to human erythrocytes. Each of the three fragments of the PfTrx-like-mero protein was separately incubated with human erythrocytes, and the adhesion was detected by Western blotting. GST protein (lane IV) was used as a control. Only fragments I and III showed binding activity to human erythrocytes, while the second fragment (fragment II) and the GST control did not. (C) The PfTrx-like-mero protein PF3D7_1104400-specific antibodies inhibited parasite invasion. The bar chart shows that the inhibitory effect on erythrocyte invasion by the specific antibodies (Ab) to the three protein fragments (I, II, and III) as well as MSP-1-42 antibodies occurs in a concentration-dependent manner. Antibodies from healthy rabbits were used as a negative control and did not show inhibitory effects.

In order to determine the role of the PfTrx-like-mero protein in merozoite invasion, glutathione *S*-transferase (GST)-tagged recombinant proteins (fragments I, II, and III) (see Fig. S2A and B in the supplemental material) were separately incubated with heparin-Sepharose and erythrocytes. All of the 3 fragments of the PfTrx-like-mero protein could bind heparin-Sepharose, but the GST protein did not show any binding to heparin (Fig. 4A). However, only fragments I and III, not fragment II or the GST control, showed binding to human erythrocytes (Fig. 4B).

### The protein-specific antibodies inhibited parasite invasion *in vitro*.

Polyclonal antibodies to the 3 fragments of the PfTrx-like-mero protein, which can recognize the native protein (see Fig. S3 in the supplemental material) were prepared and tested for their ability to inhibit merozoite invasion into erythrocytes. The result showed that all antibodies inhibited merozoite invasion in a manner similar to that of anti-MSP-1-42 (MSP-1-42 being the C-terminal 42 kDa of MSP-1) antibodies (Fig. 4C). The data further suggested that the PfTrx-like-mero protein is an important parasite ligand participating in the interaction of invading merozoites with erythrocytes.

### Immunization of a recombinant PbTrx-like protein protected mice against parasite infection.

**FIG 5:**
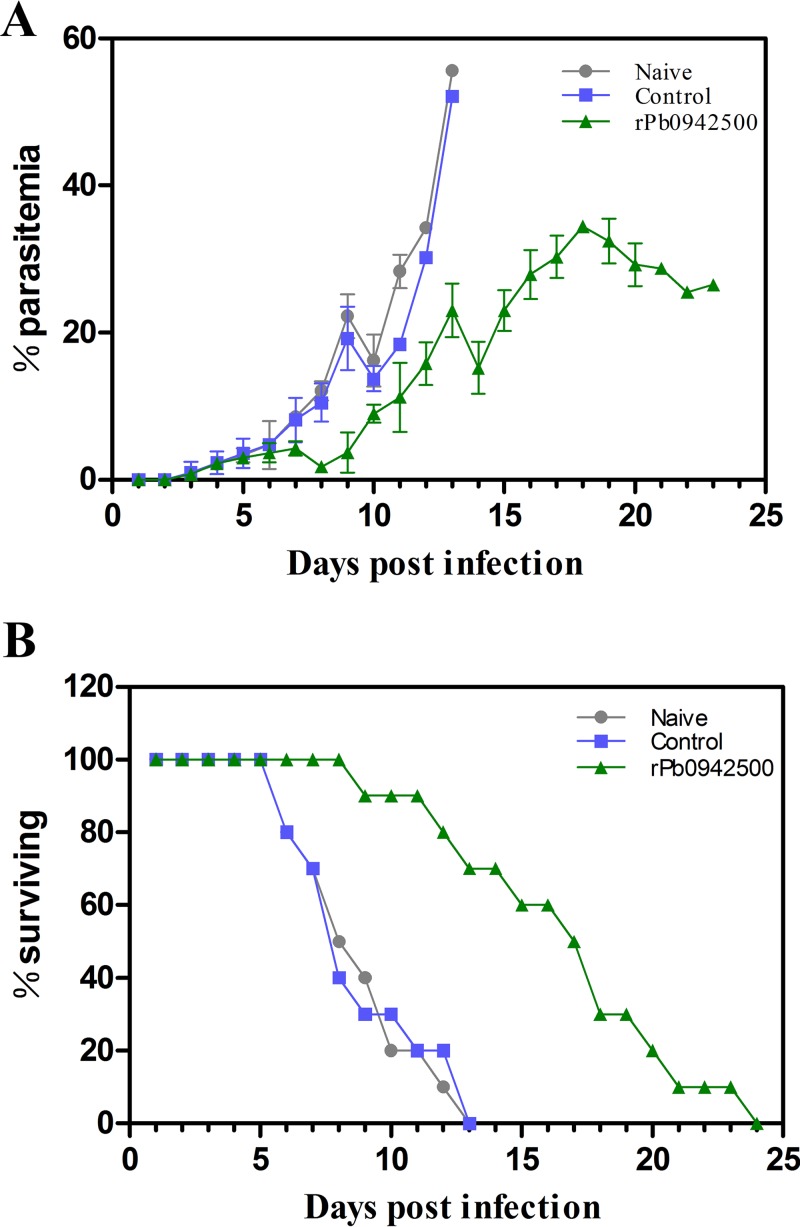
Protection against parasite infection generated by immunization with rPBANKA_0942500 in C57BL/6 mice. (A) Parasitemia variations in immunized C57BL/6 mice after challenge. The parasitemia of the mice in the naive group (without any immunization) and the control group (immunized only with Freund's adjuvant) climbed more quickly and was 2.26-fold higher at the 13th day postinfection than that of the immunized group with rPBANKA_0942500. The final parasitemia levels in each group are means for 10 mice, and the error bars indicate standard deviations (SD). (B) Survival rates of the immunized mice after challenge. The data were analyzed by the Kaplan-Meier test. Immunization of mice with rPBANKA_0942500 can prolong survival time significantly, as indicated by comparison to other groups' survival (*P* < 0.01) following challenge injection.

Both His- and GST-tagged recombinant Plasmodium berghei Trx-like proteins (PbTrx-like protein) encoded by the gene PBANKA_0942500 were generated (see Fig. S4 in the supplemental material), and the His-tagged PbTrx-like protein was used to immunize C57BL/6 mice. As shown in Fig. 5, a significant reduction in parasitemia and a prolonged survival time were observed in the immunized groups compared to the control groups after challenge with 10^6^ infected red blood cells (iRBCs). The experiment was repeated with BALB/c mice, and similar results were obtained (see Fig. S5 in the supplemental material). These data indicated that PbTrx-specific antibodies protected the immunized mice against parasite infection.

## DISCUSSION

Parasitization in erythrocytes has provided malarial parasites evolutionary advantages to evade host recognition. Erythrocytes have only basal metabolic activity and no antigen presentation pathway and are therefore ideal hiding places for the parasite to thrive. However, due to their unique function in transportation of oxygen and carbon dioxide, erythrocytes need to keep a complete antioxidative system for efficiently detoxifying the radicals and release oxidation stress. On the other hand, oxygen radicals, as components of the host innate immune system, play an important role in defending against invading pathogens, especially microbes. Antioxidation is thus critical for self-defense or self-protection. Thioredoxins (Trx), which exist in nearly all known organisms, are proteins that act as antioxidants by facilitating the reduction of other proteins by cysteine thiol-disulfide exchange. Erythrocytes contain a functional Trx system comprising Trx reductase, Trx, and at least three peroxiredoxins. Further, three Trx proteins (Trx1, -2, and -3) and 2 Trx-like proteins (Tlp1 and Tlp2) have previously been reported in P. falciparum to have antioxidation activity (26). In a recent study (13), from the heparin-binding proteome we identified a novel Trx-like protein in P. falciparum, which shows a similarity in general sequence and characteristics to Trx proteins but lacks the “-CXXC-” motif, which is the core for antioxidation function (Fig. 1). By further analysis with sequences extracted from the PlsmoDB database, a number of Trx-like proteins in P. falciparum and other malaria parasites that lack the “-CXXC-” motif but keep a general structure as Trx proteins (Fig. 1 and S1 and data not shown) were identified. The recombinant PfTrx-like-mero protein did not show any antioxidation activity, which also supported the essential role of the “-CXXC-” motif (data not show). However, the presence of this protein in all parasites in the Plasmodium genus indicates that it may possess an essential function for all Plasmodium parasites. Furthermore, we have found that the PfTrx-like-mero protein contains a GAG binding motif, which is essential for binding to the heparin sulfate-like receptor on the surface of erythrocytes, and the capacity of its binding to heparin was proven in our previous study, from which we concluded that the PfTrx-like-mero protein may play a role in invasion at the merozoite stage. However, it could not be ruled out that the PfTrx-like-mero protein may perform other functions, considering its distribution inside the erythrocyte after invasion.

A classical signal peptide sequence was predicted at the N terminus of the PfTrx-like-mero protein (Fig. 1c), which suggested that the protein may be secreted outside the parasite after expression. Real-time PCR and Western blot assays indicated that the protein is constantly expressed throughout the erythrocytic stages (Fig. 2). However, IFA and IEM results clearly showed that this protein is expressed on the surface of merozoites overlapping the MSP-1 protein (Fig. 3). The pattern of IEM is different from that of immunofluorescence, which is most likely due to technical reasons. Antigens on the merozoite surface might be lost during sample processing. Since the protein was initially identified by heparin binding with proteins extracted from late-stage parasites (13), its surface location strongly indicated that it may participate in the erythrocyte invasion process.

The involvement of the PfTrx-like-mero protein in merozoite invasion was confirmed in several analyses. First, GAG binding motifs were identified in the molecule, and the recombinant proteins indeed bound heparin, which supported the previous heparin-based affinity purification and proteomic analysis results. Importantly, the N-terminal region containing the Trx domain as well as the full-length protein bound human erythrocytes, but the C-terminal region did not (Fig. 4A and B), suggesting that the erythrocyte-binding activity was mediated by the N-terminal Trx domain. However, antibodies against three proteins (fragments I, II, and III [Fig. 1]) showed similar inhibitory effects on invasion by merozoites (Fig. 4C), indicating that the C-terminal region was functionally essential to the molecule during merozoite invasion.

To further prove that the PfTrx-like-mero protein is involved in parasite invasion, a homologous protein (Fig. S1) of P. berghei, encoded by PBANKA_0942500, was used to immunize C57BL/6 and BALB/c mice. All immunized mice showed significant protection against parasite challenge, whereas the mice in the control group were not protected at all (Fig. 5 and S5). The data collectively suggested that the novel Trx-like protein of P. falciparum is a merozoite-associated molecule participating in erythrocyte invasion.

In summary, we have identified and characterized a novel thioredoxin-like protein in P. falciparum, which was expressed on the surface of merozoites and had an erythrocyte-binding property. Specific antibodies to the molecule displayed significant invasion inhibition effects. Further, mice immunized with the homologous protein showed significant protection against parasite infection. The data facilitate the understanding of the complexity of P. falciparum erythrocyte invasion mechanism and malaria vaccine development.

## MATERIALS AND METHODS

### Ethical statement.

All procedures performed on the animals (mice and rabbits) in this study were conducted according to the animal husbandry guidelines of Jilin University. Study protocols have been reviewed and approved by the Experimental Animal Committee and the Ethical Committee of Jilin University, Changchun, China.

### Parasites.

P. falciparum strain 3D7 was cultured according to standard methods with 0.25% (wt/vol) Albumax II and 5% (vol/vol) human B^+^ serum added to a buffered medium (RPMI 1640 supplemented with HEPES, hypoxanthine, and sodium bicarbonate) with 5% hematocrit (27). The development of the parasites was synchronized with 5% (wt/vol) sorbitol. The parasites were harvested at the time points of 8, 16, 24, 32, 40, and 48 h following erythrocyte invasion and were preserved at −80°C before use.

The P. berghei strain ANKA parasites were obtained by infection of C57BL/6 and BALB/c female mice by intraperitoneal injection with 1 × 10^6^ parasitized erythrocytes (28).

### Bioinformatic analysis of the amino acid sequence encoded by PF3D7_1104400.

In our earlier study (13), a protein encoded by the PF3D7_1104400 gene sequence was identified. The whole sequences of the gene and the encoded protein (PfTrx-like-mero) were obtained from PlasmoDB (http://plasmodb.org/), and the signal peptide and transmembrane domain were predicted via the SignalP 4.1 server and TMHMM server v.2.0, respectively (29, 30). The conserved domain of PfTrx-like-mero was identified by Basic Local Alignment Search Tool (BLAST) (31), and the glycosaminoglycan (GAG) binding motifs (XBBXBX and XBBBXXBX) were determined as described previously (12).

The amino acid sequence of the PfTrx-like-mero protein was first aligned with homologues in other Plasmodium spp. (including P. reichenowi, P. berghei, P. knowlesi, P. vivax, P. chabaudi, and P. yoelii) via the multiple sequences alignment tool provided by DNAman and subsequently compared with Trx domain-containing proteins in Homo sapiens, mouse, Drosophila melanogaster, Saccharomyces cerevisiae, Arabidopsis thaliana, and Schistosoma japonicum to determine the functional sites of the Trx domain.

### Transcription analysis of the PF3D7_1104400 gene with real-time quantitative PCR.

Briefly, RNA of synchronized parasites in the 6 time points of the blood stage was extracted with TRIzol solution according to the manufacturer's instructions (Invitrogen, CA, USA). After removing the DNA remnant by DNase I (TaKaRa, Dalian, China) treatment, reverse transcription was carried out immediately in the system containing an oligo(dT) primer and reverse transcriptase (32). RT-qPCR with cDNA template and specific primers (forward, 5′-CCC ATA CAA AAG AAT CAG ATA TGC-3′; reverse, 5′-GGG TCT TGT ATG AAT TCT GG-3′) was performed in the 7500 real-time PCR system (Applied Biosystems, USA) using SYBR Premix *Ex Taq* (TaKaRa, Dalian, China). The data were analyzed by the 2^−ΔΔ*CT*^ method (where *C_T_* is threshold cycle) (33), whereby the amount of target RNA was compared to that of an internal control gene, encoding seryl-tRNA synthetase (PF3D7_1205100), which is stably expressed during the erythrocytic stage of the parasite (34). The transcription levels were determined as the means of results from the three repeated experiments, and the error bars in the figures represent the standard errors of the means (SEM).

### Expression of recombinant proteins and preparation of polyclonal antibodies.

Based on the positions of GAG binding motifs and Trx domain in the sequence, the PfTrx-like-mero protein was expressed in three fragments (fragment I, PF3D7_1104400-N888; fragment II, PF3D7_1104400-C522; and fragment III, PF3D7_1104400-1101, as illustrated in Fig. 1). His-tagged and GST-tagged recombinant proteins of the three fragments were expressed separately for obtaining polyclonal antibodies and further functional analysis. Briefly, the gene fragments encoding the three regions (regions I, II, and III) of the protein were amplified with specific primers by PCR (I, forward, 5′-gaattc ACT TCG TCC TTA CTA GAA ACC-3′, and reverse, 5′-ctcgag TAA GGA GAG TAA CAT GTC TAT TTC-3′; II, forward, 5′-ggatcc TCT GAA ACT TTT GTC CTA GG-3′, and reverse, 5′-ctcgag CAA TTC ATC TTT TTC ATT TGC TTT-3′; III, forward, 5′-ggatcc AAG TTT AGG AAT GAA AAA GTG T-3′, and reverse, 5′-ctcgag TGC GTT TGG GTC TTG TAT GA-3′), where bases in lowercase indicate restriction enzyme recognition sequences. The amplicons were separately cloned into pET-28a and pGEXT-4T-1 expression vectors. The recombinant plasmids were expressed in Escherichia coli BL21(DE3) (35, 36), and both His- and GST-tagged recombinant proteins were purified using His GraviTrap affinity columns (GE Healthcare) and glutathione-Sepharose 4B (GE Healthcare) according to the manufacturer's instructions. SDS-PAGE and Western blotting were used to evaluate the purified recombinant proteins. Polyclonal antibodies against the three fragments (I, II, III) of PF3D7_1104400 were prepared by immunization of rabbits (strain New Zealand White) with His-tagged recombinant proteins emulsified with Freund's adjuvant. The rabbits were immunized subcutaneously four times at 2 weeks' intervals, and the antisera were collected 10 days after the fourth immunization. IgG was purified from the immune sera using protein A Sepharose 4 Fast Flow (GE Healthcare), and the specificity and quality of the antibodies against the natural protein were verified by Western blotting as previously described (32).

### Expression and localization analysis of the protein in parasites by Western blotting, IFA, and IEM.

The parasites collected at the 6 time points following erythrocyte invasion were dissolved in SDS-PAGE loading buffer (250 mM Tris, 1.92 M glycine, and 1% SDS), run on SDS-PAGE gel, and transferred to a 0.2-μm nitrocellulose membrane (Bio-Rad, CA, USA) using a semidried blotting system (Bio-Rad, CA, USA) under constant 24-V voltage. After being blocked with 5% skim milk (Sigma, St. Louis, MO, USA) for 1 h at 37°C, the membrane was incubated in Tris-buffered saline with Tween 20 (TBST) containing the rabbit anti-PfTrx-like-mero-1101 IgG (1:1,000 dilution) for 12 h at 4°C. Afterwards, the membrane was washed 4 times with TBST buffer and further incubated with horseradish peroxidase (HRP)-conjugated goat anti-rabbit IgG (H+L) (Abcam, Shanghai, China) (1:3,000 dilution). The membrane was developed after washing with an enhanced chemiluminescence (ECL) reagent kit (Thermo) with a LAS 4000 mini-luminescent image analyzer (GE Healthcare) (37).

To localize the expression of the PfTrx-like-mero protein, thin blood smears of infected red blood cells (iRBCs) at the late schizont stage and free merozoites were fixed with cold methanol in −80°C for 5 min and air dried. The smears were washed three times using sterilized phosphate-buffered saline (PBS) and blocked in 5% skim milk (Sigma, St. Louis, MO, USA) for 1 h at 37°C, and the slides were incubated in PBS containing a rabbit anti-PfTrx-like-mero protein polyclonal antibody (1:50 dilution) as well as a rat anti-MSP-1-42 antibody (1:100 dilution) for 12 h in 4°C. The slides were washed with PBS and further incubated with Alexa Fluor 488-conjugated goat anti-rabbit IgG (1:1,000; Life Technologies) and Alexa Fluor 594 goat anti-rat IgG (Life Technologies) at 37°C for 1 h. The parasite nuclei were stained with Hoechst (Hoechst AG, Germany) at a 1:1,000 dilution for 5 min before image capturing with a fluorescence microscope (BX 53; Olympus, Japan).

To further reveal the location of the PfTrx-like-mero protein on the parasite, immunoelectronic microscopy (IEM) was performed. Briefly, free merozoites and parasites at the late schizont stage were fixed in 0.25% glutaraldehyde and 1% paraformaldehyde for 30 min at 4°C. Then, the samples were washed, dehydrated, and embedded in LR White resin (Sigma, St. Louis, MO, USA) at 50°C for 24 h. Ultrathin sections were blocked in 3% skim milk and then incubated with rabbit anti-PfTrx-like-mero protein antibody (1:100 dilution) at 4°C overnight as described previously (38). Subsequently, these sections were incubated in goat anti-rabbit IgG conjugated to 5-nm gold particles (1:50 dilution; Sigma, St. Louis, MO, USA) at 37°C for 1 h. Eventually, samples were examined with a transmission electronic microscope (Hitachi H-7650; Japan).

### Analysis of heparin-binding activity of recombinant proteins.

The binding activity of the PfTrx-like-mero recombinant proteins to heparin was studied as previously described (27). Briefly, the GST-tagged soluble recombinant proteins (fragments I, II, and III) and GST protein in the concentration of 0.4 μM were mixed with 40 μl heparin-Sepharose and uncoupled Sepharose 4B (GE Healthcare) and incubated for 2 h at 4°C. The mixtures were centrifuged and washed 3 times with cold PBS after incubation. The Sepharose pellets were mixed with loading buffer for SDS-PAGE and Western blotting and a GST-specific monoclonal antibody (Sungene Biotech, Tianjin, China) to determine the binding activity of the recombinant proteins with heparin.

### Adhesion of recombinant proteins to human erythrocytes.

To investigate the erythrocyte-binding activity of the PfTrx-like-mero protein, 10 μl human erythrocytes was mixed with 0.4 μM the GST-tagged recombinant proteins (fragments I, II, III) and incubated for 2 h at 4°C as described previously (5). GST protein with equal molarity was incubated with erythrocytes as a negative control, and 10 μl erythrocytes alone was used as a blank. After incubation, the RBCs were washed 3 times with PBS. Subsequently, the erythrocytes were mixed with loading buffer and boiled for SDS-PAGE. Western blotting was performed with an anti-GST monoclonal antibody as previously described.

### Invasion inhibition assay *in vitro*.

The invasion inhibition activity of the PfTrx-like-mero protein-specific antibodies was tested *in vitro* as previously described (39). Briefly, synchronized parasites at the early ring stage were diluted to 0.2% parasitemia and were incubated separately with purified rabbit IgG to the three fragments of the PfTrx-like-mero protein in concentrations of 1:250, 1:150, 1:100, 1:50, and 1:10 μg/ml in 96-well cell culture plates. The rabbit anti-MSP-1-42 IgG and a healthy rabbit IgG were used as positive and negative controls. After incubation for 48 h at 37°C, the medium in each well containing the corresponding IgG was changed once. Parasitemia was determined by flow cytometry, and 50,000 cells were analyzed in each experiment. The average parasitemia was determined after 3 repeated experiments. The invasion inhibition efficiency of the negative-control group was set as 0%, and the inhibition efficiencies of the PfTrx-like-mero protein-specific IgGs and that of the MSP-1-42-specific IgG were calculated relative to the parasitemia of the negative-control group. The results were calculated from 3 independent experiments; error bars represent SEM. One-factor analysis of variance (ANOVA) was used in this study; asterisks indicate significant differences between groups (*, *P* < 0.05; **, *P* < 0.01).

### Immunization and challenge experiments.

For immunization and challenge experiments, first, the recombinant PbTrx protein (encoded by PBANKA_0942500) of the P. berhgei ANKA strain, homologous to the PfTrx-like-mero protein (Fig. S1), was expressed and purified as described above. In the first immunization, each C57BL/6 or BALB/c mouse in the immunization group (*n* = 10) was intramuscularly injected with 50 μg His-tagged recombinant protein (rPBANKA_0942500-His) emulsified with complete Freund's adjuvant. For the following three immunizations, the mice were injected with recombination protein emulsified with incomplete Freund's adjuvant at 2-week intervals. Mice of the control group were injected only with Freund's adjuvant, and the naive group was also set without any immunization (*n* = 10 in each group). The antibody titers were determined by indirect enzyme-linked immunosorbent assay (ELISA) after 4 immunizations. Subsequently, each mouse was challenged intraperitoneally with 10^6^ iRBCs (38). The parasitemia of the mice was measured daily by counting 3,000 cells per blood smear stained by Giemsa. SPSS 19.0 and one-factor ANOVA were used in this study. Means ± standard deviations (SD) were used to express the data. The Kaplan-Meier/log rank test was used to assess the survival data between the groups.

## Supplementary Material

Supplemental material
