# Supplementary material for: A Thioredoxin Homologous Protein of Plasmodium falciparum Participates in Erythrocyte Invasion
Source: Infect Immun. 2018 Jul 23;86(8):e00289-18. doi: 10.1128/IAI.00289-18 (PMC6056854; doi:10.1128/IAI.00289-18)
Supplement: Supplemental material [file supp_86_8_e00289-18__index.html]

Supplemental material 

# A Thioredoxin Homologous Protein of Plasmodium falciparum Participates in Erythrocyte Invasion

## Supplemental material

- Supplemental file 1 -

  Fig. S1. Phylogenetic analysis of the thioredoxin family proteins. Fig. S2. The expression and purification of recombinant proteins of the three fragments of PfTrx-like protein (PF3D7\_1104400). Fig. S3. The specificity of the antibodies recognizing PF3D7\_1104400 and PBANKA\_0942500. Fig. S4. Expression and purification of recombinant PBANKA\_0942500 proteins. Fig. S5. Protection against parasite challenge by immunization with rPBANKA\_0942500 in BALB/c mice.

  PDF, 550K
