# Supplementary material for: A Thioredoxin Homologous Protein of Plasmodium falciparum Participates in Erythrocyte Invasion
Source: Infect Immun. 2018 Jul 23;86(8):e00289-18. doi: 10.1128/IAI.00289-18 (PMC6056854; doi:10.1128/IAI.00289-18)

Supplemental Data

Fig S1. Phylogenetic analysis of the thioredoxin family proteins. Molecular phylogenetic relationships of the 14 amino acid sequences of the *Plasmodium* thioredoxin family. The phylogenetic tree was constructed using MEGA 6.0 and the neighbor-joining method with 1000 bootstrap replicates. The PfTrx-like protein (PF3D7_1104400) and PBANKA_0942500 were highlighted in bold letters.

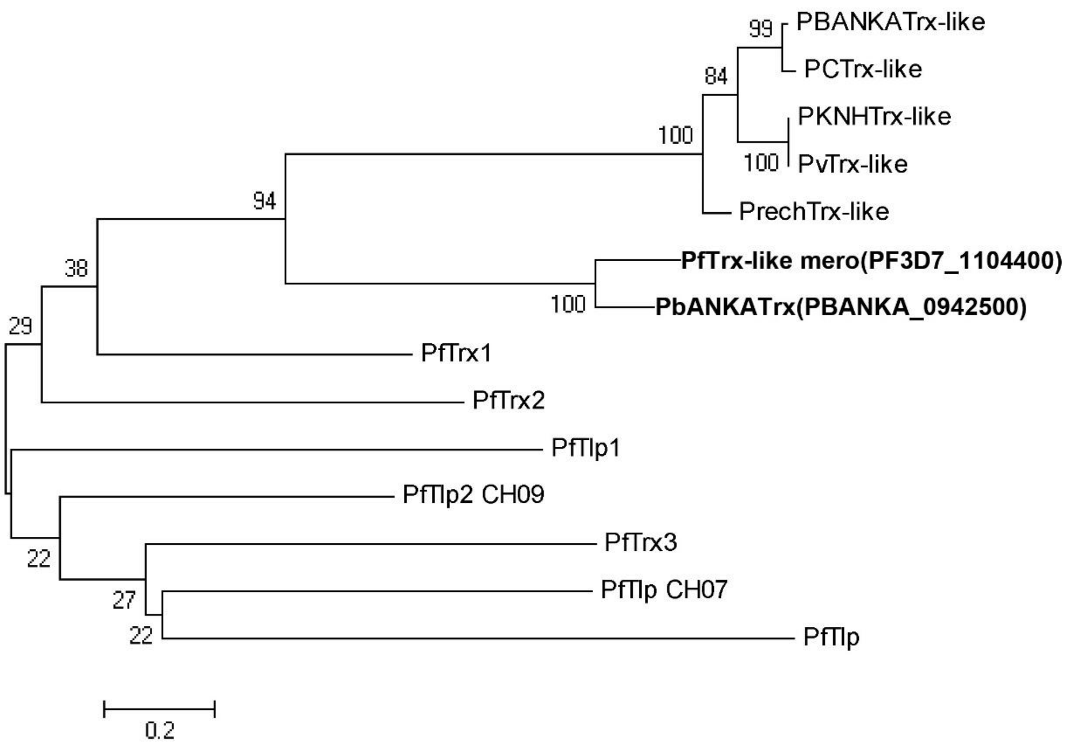

Fig. S2. The expression and purification of recombinant proteins of the three fragments of PfTrx-like protein (PF3D7_1104400). *A* and *B*, the expression and purification of GST-tagged recombinant proteins (Fragment I: PF3D7_1104400-N888; Fragment II: PF3D7_1104400-C522; and Fragment III: PF3D7 _1104400-1101). GST-tagged recombinant proteins were expressed in *E. coli*, and detected by

SDS-PAGE (*A*) and verified by Western blot (*B*). *C* and *D*, the expression and purification of His-tagged recombinant protein of three fragments were detected by SDS-PAGE (*C*) and verified by Western blot (*D*).

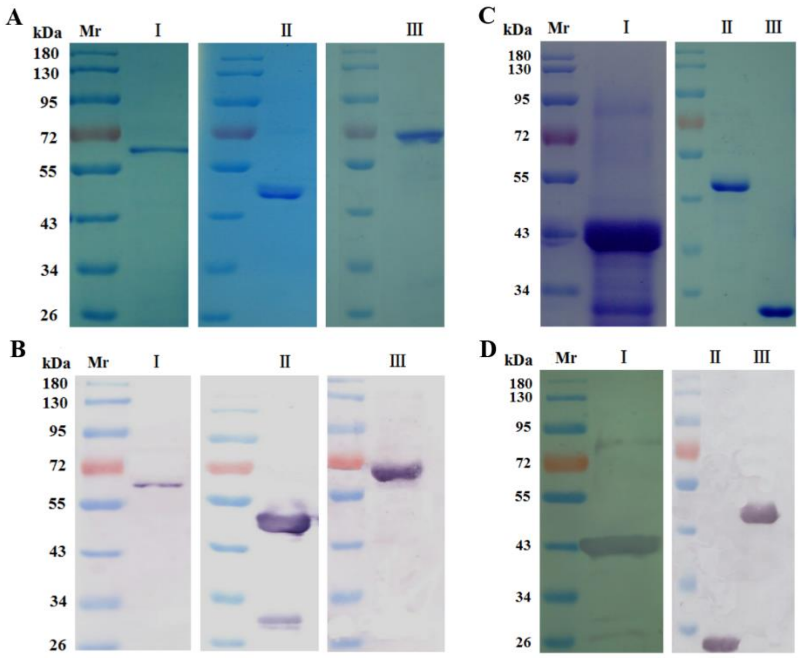

Fig. S3. The specificity of the antibodies recognizing the PF3D7_1104400 **and PBANKA_0942500. The specificity of the purified antibodies against PF3D7_1104400 and PBANKA_0942500 were verified by Western blot, the purified antibodies (1:500) were used as first antibody for examining the natural proteins in parasite lysates.**

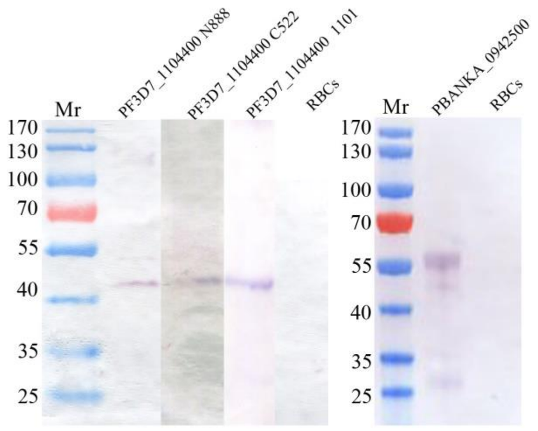

Fig. S4. The expression and purification of recombinant PBANKA_0942500 proteins. His-tagged and GST-tagged rPBANKA_0942500 (abbreviated as rPb0942500-His and rPb0942500-GST) were expressed and purified for mice immunization and antibody detection, and the purified recombinant proteins were verified by SDS-PAGE (*A*) and Western blot (*B*).

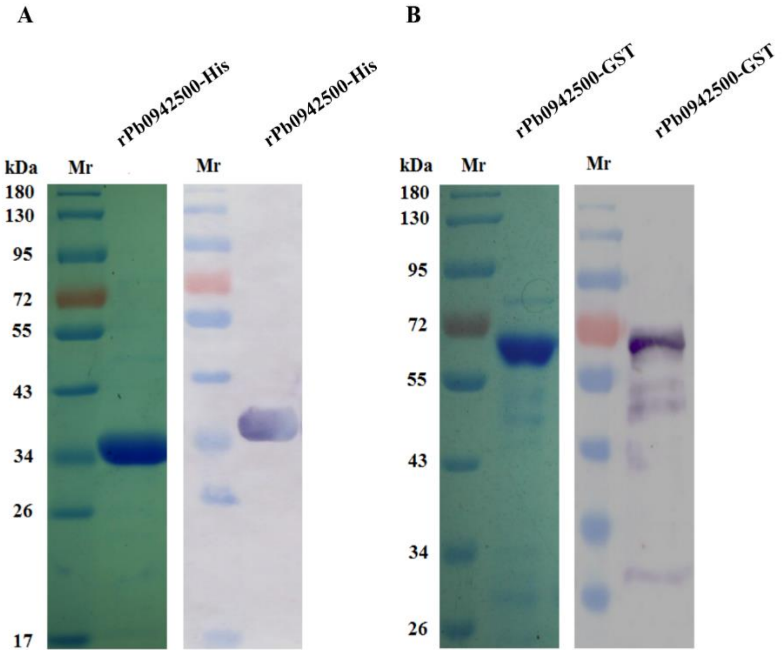

Fig. S5. The protection against parasite challenge by immunization with rPBANKA_0942500 in BALB/c mice. The parasitemia variations (*A*) and the survival rates (*B*) of the immunized BALB/c mice after challenge were shown. The parasitemia of mice in the naïve and control group climbed quickly and were 2.64-fold higher at the 13th day post infection than that of the rPBANKA_0942500-immunized group. The final parasitemia in each group are means of ten mice and the error bars represent standard deviation (SD). Mice in the immunized group survived 9 days longer than those in the two control groups.

a

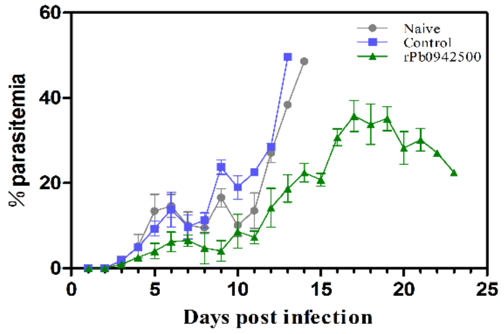

b

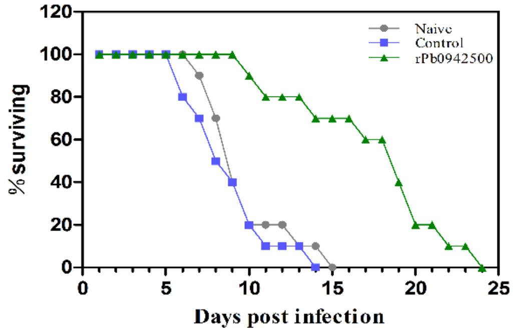

Supplement: Supplemental material [file IAI.00289-18_zii999092492s1.pdf]
